# Imaging Cellular Inorganic Phosphate in *Caenorhabditis elegans* Using a Genetically Encoded FRET-Based Biosensor

**DOI:** 10.1371/journal.pone.0141128

**Published:** 2015-10-20

**Authors:** Swayoma Banerjee, Wayne K. Versaw, L. Rene Garcia

**Affiliations:** 1 Department of Biology, Texas A&M University, 3258 TAMU, College Station, TX, 77843-3258, United States of America; 2 Howard Hughes Medical Institute, College Station, TX, 77843-3258, United States of America; East Carolina University, UNITED STATES

## Abstract

Inorganic phosphate (Pi) has central roles in metabolism, cell signaling and energy conversion. The distribution of Pi to each cell and cellular compartment of an animal must be tightly coordinated with its dietary supply and with the varied metabolic demands of individual cells. An analytical method for monitoring Pi dynamics with spatial and temporal resolution is therefore needed to gain a comprehensive understanding of mechanisms governing the transport and recycling of this essential nutrient. Here we demonstrate the utility of a genetically encoded FRET-based Pi sensor to assess cellular Pi levels in the nematode *Caenorhabditis elegans*. The sensor was expressed in different cells and tissues of the animal, including head neurons, tail neurons, pharyngeal muscle, and the intestine. Cytosolic Pi concentrations were monitored using ratiometric imaging. Injection of phosphate buffer into intestinal cells confirmed that the sensor was responsive to changes in Pi concentration *in vivo*. Live Pi imaging revealed cell-specific and developmental stage-specific differences in cytosolic Pi concentrations. In addition, cellular Pi levels were perturbed by food deprivation and by exposure to the respiratory inhibitor cyanide. These results suggest that Pi concentration is a sensitive indicator of metabolic status. Moreover, we propose that live Pi imaging in *C*. *elegans* is a powerful approach to discern mechanisms that govern Pi distribution in individual cells and throughout an animal.

## Introduction

Inorganic phosphate (Pi) is a component of nucleic acids and phospholipids, plays key roles in signal transduction cascades, and is a substrate for the generation of ATP via glycolysis and oxidative phosphorylation. The concentrations of Pi in different cells and both intra- and extra-cellular compartments must, therefore, be maintained within certain limits, despite fluctuations in dietary supply and metabolic demand. Multiple Pi transporters, as well as metabolic recycling and excretory activities have been identified in animals [1, 2]. However, a comprehensive understanding of their mechanisms and how these are integrated to achieve Pi homeostasis is limited by the inability to monitor Pi concentrations with spatial and temporal resolution. ^31^P-NMR has been used to estimate Pi concentrations in acidic cellular compartments, such as vacuoles, but cannot readily distinguish concentrations in the pH-neutral compartments that comprise the cytoplasm [3]. This method also lacks the cellular and temporal resolution needed to accurately measure changes in Pi levels within single cells. Novel technologies, such as biosensors, are therefore needed to study Pi dynamics in live animals.

Genetically encoded sensors, or biosensors, have proven to be effective tools for monitoring changes in the concentrations of small molecules and ions in live cells [4, 5]. Such sensor proteins typically consist of a ligand-binding domain fused to one or two spectral variants of green fluorescent protein (GFP). Ligand binding to the sensor elicits concentration-dependent changes in protein conformation that are detected by changes in fluorescence intensity, fluorescence resonance energy transfer (FRET) or fluorescence lifetime imaging microscopy (FLIM) [6–9]. Sensors can be expressed in specific cells, targeted to specific cellular locations, and because their detection is non-destructive, organisms can be monitored over time.

Previously, Gu et al, [10] constructed a series of genetically encoded FRET-based Pi sensors named fluorescent indicator protein for inorganic phosphate (FLIPPi). FLIPPi sensors consist of a Pi binding protein (PiBP) derived from cyanobacteria *Synechococcus sp*., translationally fused between enhanced cyan fluorescent protein (eCFP) and enhanced yellow fluorescent protein (eYFP). One FLIPPi variant with an *in vitro* dissociation constant (K_d_) for Pi of 30 mM, FLIPPi-30m, was expressed in cultured animal cells to monitor cytosolic Pi. Changes in FRET indicative of altered cytosolic Pi concentrations were detected in Pi-starved CHO cells when treated with exogenous Pi, and also in COS-7 cells that co-expressed the human Na^+^/Pi co-transporter, Pit2 [10].

Recently, Mukherjee et al, [11] modified a FLIPPi sensor to generate second-generation Pi sensors with greater dynamic range and binding affinities optimized for *in vivo* studies. Substitution of the eYFP portion of a FLIPPi sensor with a circularly permuted Venus, a pH- and chloride-insensitive version of YFP [12, 13], enhanced the dynamic range of the Pi-dependent FRET response. The resulting circularly permuted sensor was named cpFLIPPi. Mutagenesis of the PiBP component of cpFLIPPi yielded sensors with *in vitro* K_d_ values ranging from 80 μM to 11 mM. Cytosol- and plastid-targeted forms of the cpFLIPPi-6.4m sensor (K_d_ of 6.4 mM) were expressed in *Arabidopsis thaliana*. Live imaging with these transgenic plants revealed reversible changes in cytosolic Pi concentrations in response to Pi starvation and differences in the accumulation of Pi in plastids caused by Pi transporter mutations [11].

In this study, we demonstrate the utility of live Pi imaging in a model animal, *Caenorhabditis elegans*. Ratiometric FRET analyses revealed changes in the concentrations of Pi in the cytosol of different cells and tissues during development and in response to environmental and metabolic cues, including nutrient starvation and exposure to a metabolic inhibitor. These results suggest that cytosolic Pi is a sensitive indicator of cellular metabolic status and that live imaging can be an effective approach to identify novel effectors of Pi homeostasis.

## Materials and Methods

### Strains and culture methods

Strains were maintained at 20°C on NGM agar plates and fed with *E*. *coli* OP50. Alleles used in the study were: *pha-1(e2123)* LGIII [14], *him-5(e1490)* allele LGV [15] and *lite-1(ce314)* LGX [16]. Transgenic strains include: *rg*Ex682 [P*gtl-1*:*FLIPPi-6*.*4m*]; *him-5 (e1490)*, *rg*Is13 [integrated P*gtl-1*:*FLIPPi-6*.*4m*]; *pha-1(lf)*, *him-5(e1490)*, *lite-1(lf)*, *rg*Ex730[P*hsp-16*:*FLIPPi-6*.*4m*+ pBX1];*pha-1(lf)*, *him-5(e1490)*, *lite-1(lf)*, *rg*Ex725[P*hsp-16*:*Pi binding protein-6*.*4m*::cpVenus + pBX1].

### Plasmids

Construction of cpFLIPPi-6.4m was previously described [11]. In brief, cpFLIPPi-6.4m is a chimeric protein in which the cyanobacterial Pi binding protein [10] is sandwiched between eCFP and circular permuted Venus. The K_d_ for Pi binding to the purified sensor protein is 6.4 mM when assayed at pH 7.5. In this work, cpFLIPPi-6.4m was translationally fused to the first 91 amino acids of the beta integrin protein PAT-3 [17]. This region of PAT-3 targets cpFLIPPi-6.4m to the cytoplasmic side of the cell membrane, as well as labeling intracellular perinuclear membranes. The sensor was targeted to the cell membrane to minimize the effects of different promoters on protein accumulation and to restrict the sensor to the desired cells. This targeting also facilitated imaging because small amounts of the sensor were more readily detected when localized to specific regions of the cell.

pPD122-39 (Addgene), a vector from the Fire plasmid collection [18], was used as the source for the membrane targeting sequence. The plasmid contains the PAT-3 membrane localization sequence (MLS) fused to GFP and the *unc-54* 3' UTR. The GFP sequence was removed from the plasmid using inverse PCR and the primers 5'-tagcattcgtagaattccaactgagcgccg and 5'-tttttctaccggtacctcggatctatcatgaag. A 2.6 kb *BamH*1-*Hind*III fragment containing cpFLIPPi-6.4m was cut from pRSET/cpFLIPPi-6.4m [11] and ligated to the GFP-deleted PAR-3 MLS plasmid. The gateway ATTR cassette C.1 (Invitrogen, CA) was then ligated to a *Sma*1 site to create the destination plasmid pLR318. The 1.6 kb ATTL-flanked intestinal *gtl-1* promoter, contained in the plasmid pBL63 [19] was then recombined with pLR318, using LR clonase (Invitrogen) to generate the plasmid pLR316. The *hsp-16* heat shock promoter was PCR-amplified from genomic DNA using the primers: 5'-ggggacaagtttgtacaaaaaagcaggcttaagcttgcatgcctgcagg and 5'-ggggaccactttgtacaagaaagctgggtgctagccaagggtcctcct. The 540 bp ATTB-flanked *hsp-16* heat shock promoter was then recombined with the plasmid pDG15, using BP clonase (Invitrogen) to generate the plasmid pBL172. The ATTBL-flanked *hsp-16* promoter in pBL172, was then recombined with pLR318, using LR clonase, to generate the plasmid pLR323. To determine how much 542 nm emission from cpFLIPPi-6.4m was due to direct excitation of cpVenus by the 445 nm laser, the eCFP sequence from pLR323 was removed using inverse PCR and the phosphorylated primers: 5'-gggatcggtaccgtaggatttctaacagcgacctcggctcaagccc and 5'-gcggcccggatctttttctaccggtacctcggatctatcatgaag. The plasmid was religated to generate pLR324.

### Transgenics

To generate the *rg*Ex682 [P*gtl-1*:cp*FLIPPi-6*.*4m*] transgenic line, a mixture of 50 ng/μl of pLR316 and 150 ng/μl of pUC18 was injected into the germline of N2 hermaphrodites [20]. Stable transmitting lines expressed cpFLIPPi-6.4m in the intestines from an extrachromosomal array. To integrate the extrachromosomal *rg*Ex682 array into the genome, transgenic hermaphrodites were treated with 30 μg/ml trimethylpsoralen for 30 minutes and exposed to 340 μW/cm^2^ UV for 1 minute [21] to create the line *rg*Is13 [integrated P*gtl-1*:cp*FLIPPi-6*.*4m*]. Two factor genetic mapping placed the integrated P*gtl-1*:cp*FLIPPi-6*.*4m* on chromosome I. To generate *rg*Ex730 [P*hsp-16*:cp*FLIPPi-6*.*4m*+ pBX1] and *rg*Ex725 [P*hsp-16*:*Pi binding protein-6*.*4m*::cpVenus +pBX1] transgenic lines, DNA mixtures containing 50 ng/μl of pLR323 or pLR324, 50 ng/μl of pBX1 (*pha-1* plasmid) and 100 ng/μl pUC18 were injected into the germline of *pha-1(lf)*, *him-5(e1490)*, *lite-1(lf)* hermaphrodites. *pha-1*-rescued transformants, which can stably propagate at 20°C, were then selected.

### Live imaging and FRET analysis of cytosolic Pi

Worms expressing the cpFLIPPi-6.4m sensor were imaged to measure Pi-dependent FRET in the intestine, head neurons, tail neurons and pharyngeal muscle. Worms were transferred to a glass slide with a thin layer of 8% noble agar, immobilized with 25 mg/ml Polybead polystyrene 0.1 μm microspheres (Polysciences, Inc., WA) [22] and covered with a glass cover slip prior to imaging. Hermaphrodites were developmentally staged based on the cell division patterns of the vulval P5.p, P6.p and P7.p cell descendants. Mounted animals were analyzed on an inverted Olympus IX81 microscope equipped with a Yokogawa CSU-X1 Spinning Disk confocal unit and an iXon897 EMCCD camera (Andor Technology, USA). Animals were viewed with 10x (0.3 n.a) or 40x (oil-immersion 1.3 n.a.) objectives. The laser wavelength used for CFP excitation was 445 nm. The emission filter for CFP was 483/32 nm and the emission filter for FRET was 542/27 nm. The laser wavelength used for direct excitation of cpVenus was 515 nm. The same laser excitation and image acquisition settings (in Metamorph software) were used for all experiments: 40% laser power for the 445 nm laser, 10% laser power for the 515 nm laser, 1000 ms exposure time, electron multiplier gain set at 13%, and pre-amplifier gain set at 5%. Image analysis was done using ImageJ software and statistics were conducted using Graphpad Prism 5 (version 4.03) software. Regions of interest were drawn across the image and mean intensity values were measured in the CFP, FRET and cpVenus emission channels. FRET intensity values were corrected for donor spectral bleedthrough, which was determined by acceptor photobleach. To photobleach cpVenus, a 100x oil-immersion (1.35 n.a.) objective was used to focus on a section of the intestine, and a 515 nm excitation laser was then used at maximum intensity. FRET ratios were calculated as the corrected FRET emission (445 ex: 542 em) divided by the eCFP emission (445 ex: 483 em).

Comparative analysis of neurons, pharyngeal muscles and intestine was carried out by imaging the animals with a 40x (oil-immersion, 1.3 n.a.) objective. To correct for any possible alteration in the FRET/eCFP values due to changes in image acquisition between the 10x (0.3 n.a) and 40x (oil-immersion 1.3 n.a.) objectives, the intestine of the same worm was imaged sequentially using the two camera acquisition settings to calculate FRET/eCFP ratio. Mean FRET/eCFP values were calculated from the same region of the intestine imaged under 10x and 40x. The difference between the mean FRET/eCFP ratios was used as an additive correction factor for all subsequent experiments carried out using the 40x objective.

Sodium cyanide treatment differentially reduced eCFP and cpVenus fluorescence intensities of cpFLIPPi-6.4m when the sensor was expressed in worms, but not when treated *in vitro*. We attribute this effect to undefined changes in the cellular environment. Because the effect of cyanide on eCFP would influence both the numerator and denominator components of the FRET/eCFP ratio, we instead normalized corrected FRET values by emission intensity values of directly excited cpVenus (515 ex: 542 em), i.e., FRET/cpVenus. In this case, raw FRET intensities were corrected for both donor spectral bleed-through and acceptor cross-excitation. The cross-excitation correction factor was determined by imaging *rg*Ex725 [P*hsp-16*:*Pi binding protein-6*.*4m*::cpVenus + pBX1] worms, which expressed cpVenus fused to the C-terminus of the Pi binding protein.

### *In vitro* Pi binding assay

The cpFLIPPi-6.4m sensor was expressed in *E*. *coli* BL21 (DE3) and the protein was purified from 100 ml culture lysates using immobilized metal affinity chromatography. The sensor protein was dialyzed against 20 mM Tris-HCl (pH 7.5) overnight at 4°C. Protein concentration was quantified using the Bradford colorimetric assay then diluted in protein dilution buffer, 100 mM K-gluconate, 30 mM NaCl, 25 mM MES, 25 mM HEPES, 40% sucrose, pH 7.5 [11] to yield fluorescence intensity values equivalent to those detected *in vivo*. Different concentrations of potassium phosphate buffer (pH 7.5) were added to the diluted sensor then 5 μl aliquots were transferred to individual wells of a 24-well silicone isolator (Electron Microscopy Sciences, Hatfield, PA). The wells were imaged for Pi-dependent FRET using an epifluorescence–equipped Olympus BX51 upright compound microscope (Olympus, USA) with a 10x objective. Image capture settings were the same as those used for *in vivo* analyses. FRET ratio data were fit to a single-site binding isotherm to estimate dissociation constant (K_d_) values.

### Nutrient starvation and cyanide treatment

To study the effect of environmental conditions on cellular Pi levels, worms were subjected to nutrient starvation and exposure to the respiratory inhibitor sodium cyanide. Worms were grown on Nutrient Growth Media (NGM) plates seeded with a saturated culture of *E*.*coli* OP50. 24 h adult hermaphrodites containing the P*hsp16*:cpFLIPPi-6.4m sensor were heat-shocked at 32°C for 1 h to allow induction of the heat shock promoter, then returned to room temperature (22–24°C) for 3 h before imaging. For the starvation experiment, fluorescent worms were selected after heat shock, transferred to NGM plates with or without *E*.*coli* OP50, and maintained in the respective plates for 5 h. Fed and starved worms were imaged within 5–7 h of starvation. For the cyanide treatment, fluorescent worms were transferred from NGM plates to a thin layer of 8% noble agar on a glass slide, immobilized with 25 mg/ml 0.1 μm polystyrene beads (Polysciences, Inc., WA) [22] then imaged. The same worms were then treated with 2 μl of a 10 mM NaCN solution for 30 sec, then quickly covered with a glass cover slip and imaged within 1.5 to 2 min after addition of cyanide. To detect expulsion of Pi from worms, 35–40 worms were suspended in 5 μl of a 1.7 μM cpFLIPPi-6.4m protein solution in a single well of a 0.5 mm silicone isolator. The protein solution was imaged before and after addition of sodium cyanide to a final concentration of 10 mM then imaged over 5 min at 1 min intervals. The control group of worms was treated with sensor protein solution instead of sodium cyanide.

### Microinjection of inorganic phosphate

Animals were injected with phosphate buffer using a technique similar to germ line microinjections [20]. In brief, two-day old adult *rg*Is13 hermaphrodites were first immobilized with 1–2 μl of freshly made 1 M dopamine (Sigma) (dissolved in water). Even after the dopamine extensively oxidizes in air, it will still paralyze the worm in seconds. Dopamine was used as a paralytic, rather than the more commonly used sodium azide, since we did not want a mitochondrial poison to disrupt metabolism prior to, or during imaging. The paralyzed worms were placed on the surface of a 5% noble agar pad then imaged without a coverslip using an epifluorescence–equipped Olympus BX51 upright compound microscope (Olympus, USA). The animals were imaged in the open air using a 10x objective and excited with filtered 445 nm light. The 483 nm and 542 nm emissions were recorded simultaneously, using a Dual View Simultaneous Image Splitter (Photometrics, AZ) and a Hamamatsu ImagEM Electron multiplier (EM) CCD camera. After the animals were imaged, they were immediately (~30 sec to 1 min) placed in a puddle of series 700 Halocarbon oil on a coverslip containing dried 2% agarose. The animals were placed on an inverted microscope and visualized using DIC optics and a dry 40x objective (0.75 n.a). Microinjection needles, filled with different concentrations of sterile KH_2_PO_4_ buffer (~pH 6.3 to 6.5), were fit onto a hydraulic micromanipulator, and the needle was then inserted through the cuticle and into one of the intestinal cells. An Eppendorf FemptoJet pump (Eppendorf, North America) was connected to the needle holder. The intestinal cells are connected to each other via cell-specific gap junctions [23], and thus we were able to observe the injected buffer move throughout the whole intestine. The total time that the worms were kept on the injection pad was no more than 20 seconds. The needle was inserted into the dorsal side of the hermaphrodite, generally between the anterior and mid-body region. The injector was set to deliver the Pi at maximum pressure. Generally, two to three seconds was required to fill the intestine. However, when the needle was removed, liquid could be seen to leak from the intestinal puncture site. The approximate radius of the pre-injected hermaphrodite intestine was 40 microns and the approximate length was 850 microns. The amount of time required to fill the intestine was used to calculate the volume of the injected Pi buffer. Specifically, Pi buffer was injected into a puddle of halocarbon oil for the same amount of time then the diameter of the spherical aqueous droplet (~50 micrometers) was used to calculate the volume of buffer.

After the animals were injected, they were immediately (within 30 sec to 1 min) transferred to the 5% noble agar pad then reimaged in open air with the exact same microscope, camera and illumination settings used prior to the microinjection. The animals were imaged in open air because we found that putting a coverslip on the punctured animals at any time during the manipulations eventually caused them to explode. The data were analyzed using the Metamorph software (Molecular Devices, PA). The dimensions of the intestine were determined from micrographs of the injected hermaphrodites. The idealized volume of the intestine was estimated from the dimensions of the cylinder-shaped organ: πr^2^h = (3.14) (40x10^-4^)^2^(850x10^-4^) cm^3^. Assuming that the injected Pi fills the intestine, the maximum final concentration of Pi in the injected intestine would be 5.8 mM after the 50 mM injection, 11.7 mM after the 100 mM injection and 117 mM after the 1 M injection.

To monitor Pi injections in real time, 100 mM propidium iodide was added to the Pi buffer. The animals were immobilized on a dried 2% agar pad and covered with halocarbon oil. The animals were pierced with the needle and images for eCFP (445 ex/ 483 em), FRET (445 ex/ 542 em) and propidium iodide (561 ex/ 650 em) were captured using the Olympus IX81 microscope, Yokogawa CSU-X1 Spinning Disk confocal unit, iXon897 EMCCD camera set-up. A 1–2 sec injection delivered an indeterminate bolus of Pi into the worm, and then images were taken every 4 sec for 30 sec. The propidium iodide was used to confirm the location of the injection and to estimate the spread of Pi buffer. ROIs used for data analysis were applied to the images approximately 50 to 100 μm from the needle puncture site. The data were analyzed using the Metamorph software.

## Results

### Expression and ratiometric imaging of a Pi sensor in *C. elegans*

**Fig 1:**
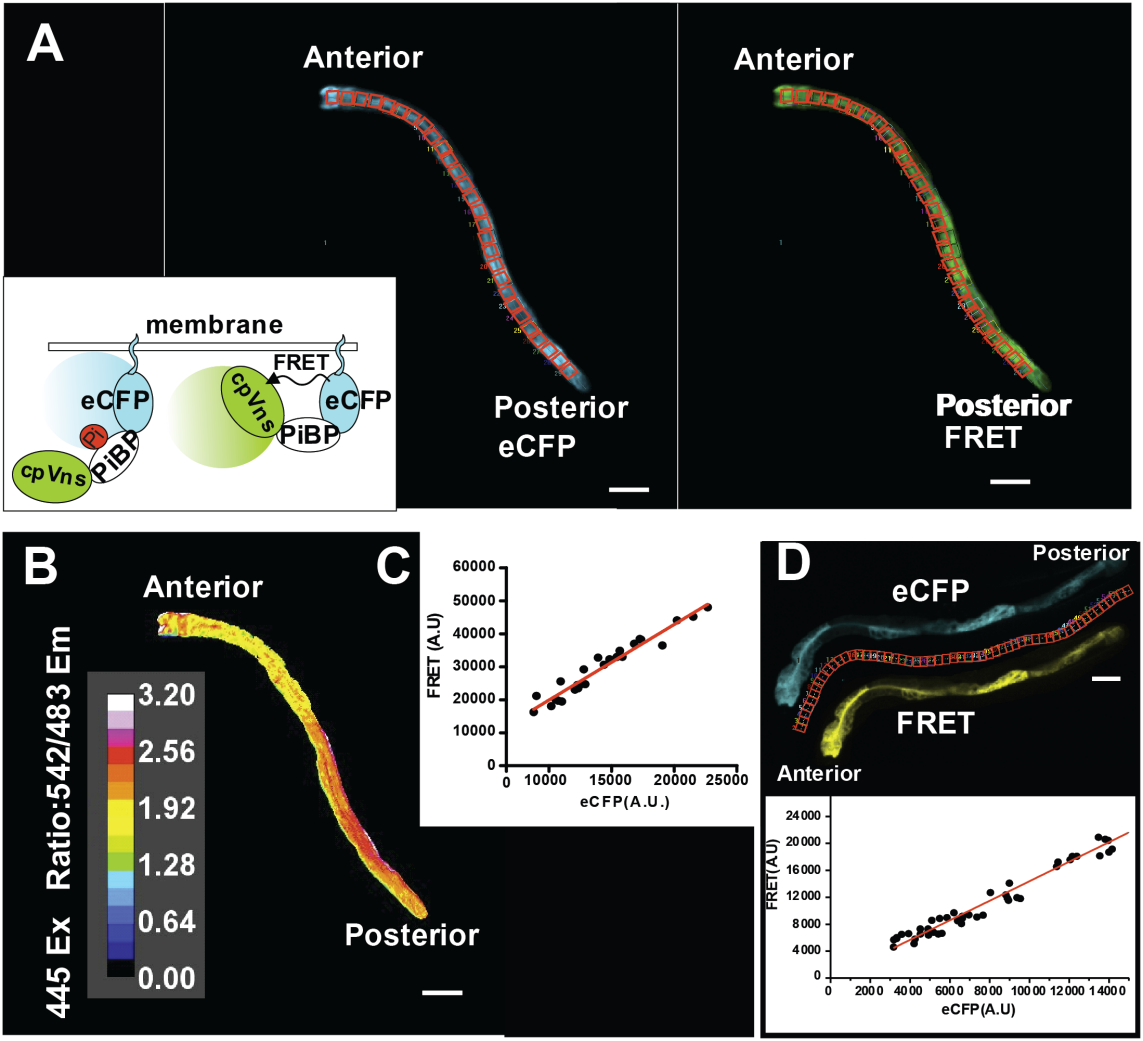
The FRET and eCFP ratio of cpFLIPPi-6.4m between intestinal cells of *C*. *elegans* is similar. (A) eCFP and FRET emission channels, respectively showing donor and non-corrected FRET emission after the donor is excited with the 445nm laser. Inset shows schematic representation of cpFLIPPi-6.4m tethered to the cytosolic face of the cell membrane. Binding of ligand (Pi) alters the orientation or distance between the donor and acceptor and decreases FRET. FRET Excitation-445 nm: Emission- 542/27 nm, eCFP Excitation- 445 nm: Emission 483/32 nm. Scale bar is 100 microns. (B) Pseudo-colored FRET ratio image was created by dividing FRET pixel intensity values by eCFP pixel intensity values. Color bar shows the range of FRET ratios in the intestine. (C) Plot of non-corrected FRET and eCFP mean intensity values obtained from arbitrary user defines ROIs. The red slope line was calculated as a linear regression (least-squares method) of the data points. Slope of the graph represents mean FRET/eCFP ratio of the intestine of a single animal. (D) The upper panel shows eCFP (top) and FRET (bottom) emission images of a 24 hr adult hermaphrodite with differential intestinal cpFLIPPi-6.4m expression. Scale bar is 100 microns. The red boxes between the images show the regions of interest (ROI) used to quantify the eCFP and uncorrected FRET emission intensities. The ROIs were superimposed on the intestinal images and used to calculate the average pixel intensity. The lower panel shows the eCFP intensities (in arbitrary units) plotted against the uncorrected FRET emission intensities for the animal in the upper panel. The red slope line was calculated as a linear regression (least-squares method) of the data points.

To assess the efficacy of live Pi imaging in *C*. *elegans*, we first needed to determine whether consistently uniform expression of the Pi sensor could be detected. In this work, we used a cytosolic membrane-targeted cpFLIPPI-6.4m (inset Fig 1A). The membrane localization aided in the imaging analysis, since it promoted the concentration of low amounts of the sensor to specific cell regions, and reduced the diffusion of the sensor in photobleaching and micro-injection experiments described later in this report. The use of a membrane-targeted sensor may lead to intermolecular FRET, but this would contribute to baseline FRET and therefore have minimal effect on measurements of changes in Pi dependent FRET. We initially expressed cpFLIPPI-6.4m in intestinal cells, because this is where Pi and other nutrients are first assimilated. Moreover, previous studies showed that intestinal Pi metabolism is critical for growth and development of *C*. *elegans* [24–26]. We predicted that cytosolic Pi, and thus the corresponding FRET signals, would be similar across the intestine because the cells are interconnected by gap junctions [27]. As shown in Fig 1A, eCFP donor emission and FRET emission from the Pi sensor were both roughly uniform throughout the intestine.

Several imaging-based methods have been described for quantitative analysis of FRET, including ratiometric approaches, e.g., FRET/donor and FRET/acceptor emission intensity ratios [28, 29], and measurement of donor fluorescence lifetime [30]. For the bulk of our studies, we chose to use FRET/donor ratiometric analysis (Fig 1B). This approach provides high sensitivity because FRET (numerator) and donor eCFP emission (denominator) both change in response to ligand concentration and the changes are in opposite direction. In this analysis, a decrease in the FRET/donor ratio indicates an increase in cytosolic Pi concentration, whereas an increase in the ratio reports the opposite.

Although expression of cpFLIPPi-6.4m appeared similar in the intestine, it was possible that differences in protein expression levels could affect FRET and/or eCFP signals. To test this possibility, we measured mean FRET and eCFP emission intensities within defined regions of interest (ROIs) that encompassed the entire intestine. Intensity values for both FRET and eCFP emission varied more than 2.5-fold at different positions in the intestine, but these were proportional over the entire range, as indicated by the plot of FRET versus eCFP in Fig 1C. We also found that within exceptional individuals, expression of the sensor can differ up to 4-fold in their intestinal cells (Fig 1D), but still exhibit a proportional relationship between eCFP and FRET emission intensities. The slope of this line represents the composite FRET/eCFP ratio for the intestine, which reflects cytosolic Pi concentration. These results indicate that *in vivo* FRET/eCFP ratio is largely insensitive to protein expression levels. However, because it remained possible that FRET/eCFP may be inconsistent at very low or very high sensor protein concentrations, we restricted our analyses to worms that exhibited fluorescence intensities that can be captured within our camera's linear range. We used same laser and camera settings for all experiments, as specified in the materials and methods.

**Fig 2:**
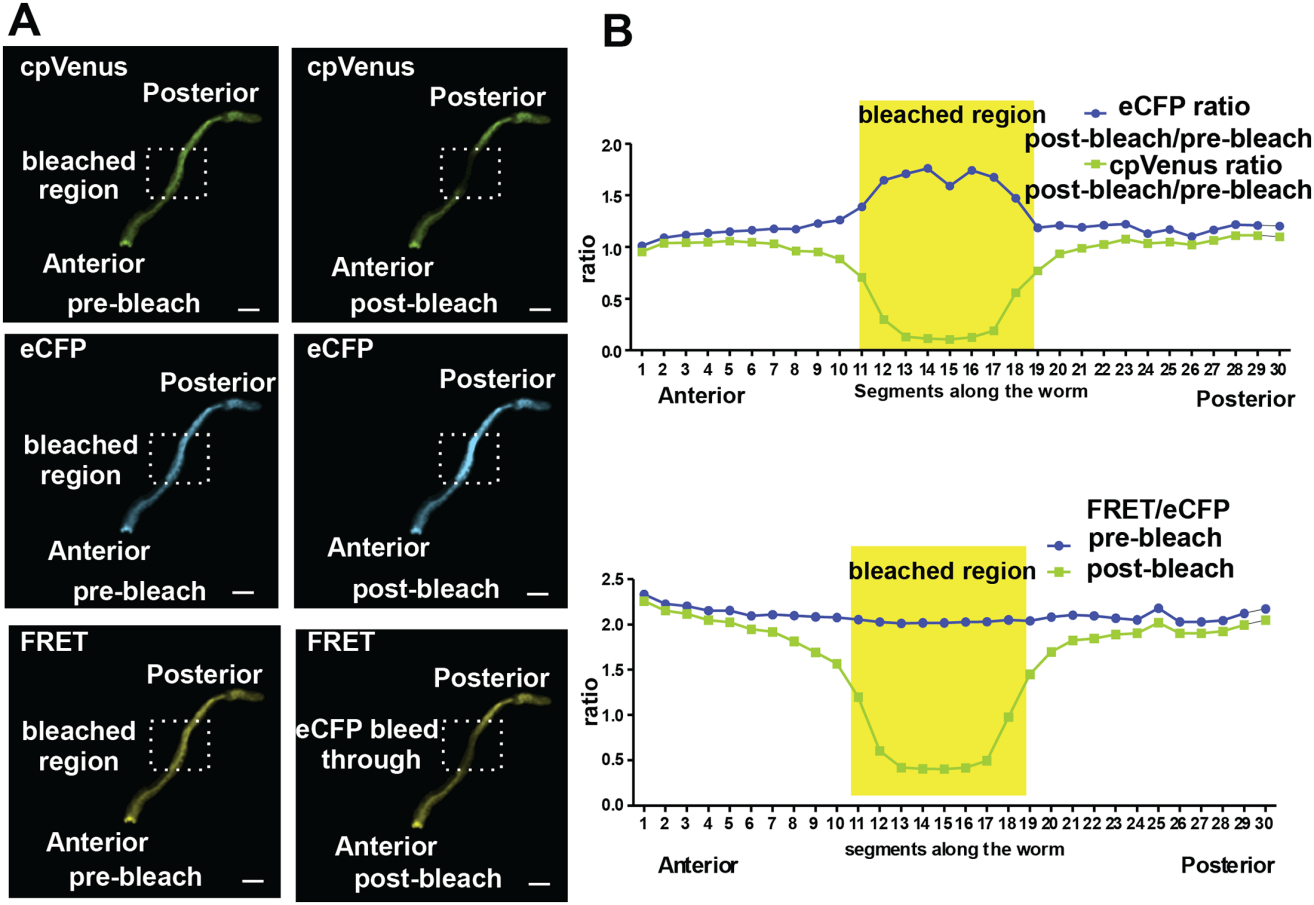
Fluorescence in the 445 excitation/540 emission channel includes approximately 30% eCFP cross emission. Percentage cross emission is calculated by photobleaching the acceptor. (A) A decrease in acceptor emission is accompanied by an increase in donor emission and a decrease in FRET emission in the acceptor-photobleached region of the intestine. (B) Graphs represent plots of eCFP emission (post bleach/pre-bleach), cpVenus emission (post bleach/pre-bleach), and FRET emission (post bleach/pre-bleach) comparatively in the bleached and non-bleached regions of the intestine. FRET Excitation-445 nm: Emission- 542/27 nm, eCFP Excitation- 445 nm: Emission 483/32 nm. Scale bar is 100 microns.

The eCFP emission spectrum partially overlaps with that of the FRET acceptor cpVenus. Consequently, some of the fluorescence attributed to FRET is due to spectral bleed-through from eCFP [31]. To quantify the fraction of FRET emission due to eCFP spectral bleed-through, we used an acceptor photobleaching approach [32]. The cpVenus component of the membrane targeted cpFLIPPi-6.4m was photobleached in a portion of the intestine and this resulted in the expected increase in eCFP emission due to dequenching of the FRET donor (Fig 2A). The same treatment was applied to eight independent worms, and pre- and post-photobleach values for eCFP and cpVenus emission and for FRET/eCFP ratios across the entire intestine were analyzed (Fig 2B). Photobleaching had no effect on fluorescent signals outside the targeted region, whereas FRET/eCFP of the bleached region was reduced to 32 ± 0.08% (n = 8) of the pre-bleached value. This remaining signal represents the fraction of FRET due to eCFP bleed-through. The bleed-through fraction was therefore removed from raw FRET signals in all subsequent experiments.

### Monitoring Pi-dependent FRET responses in live worms

**Fig 3:**
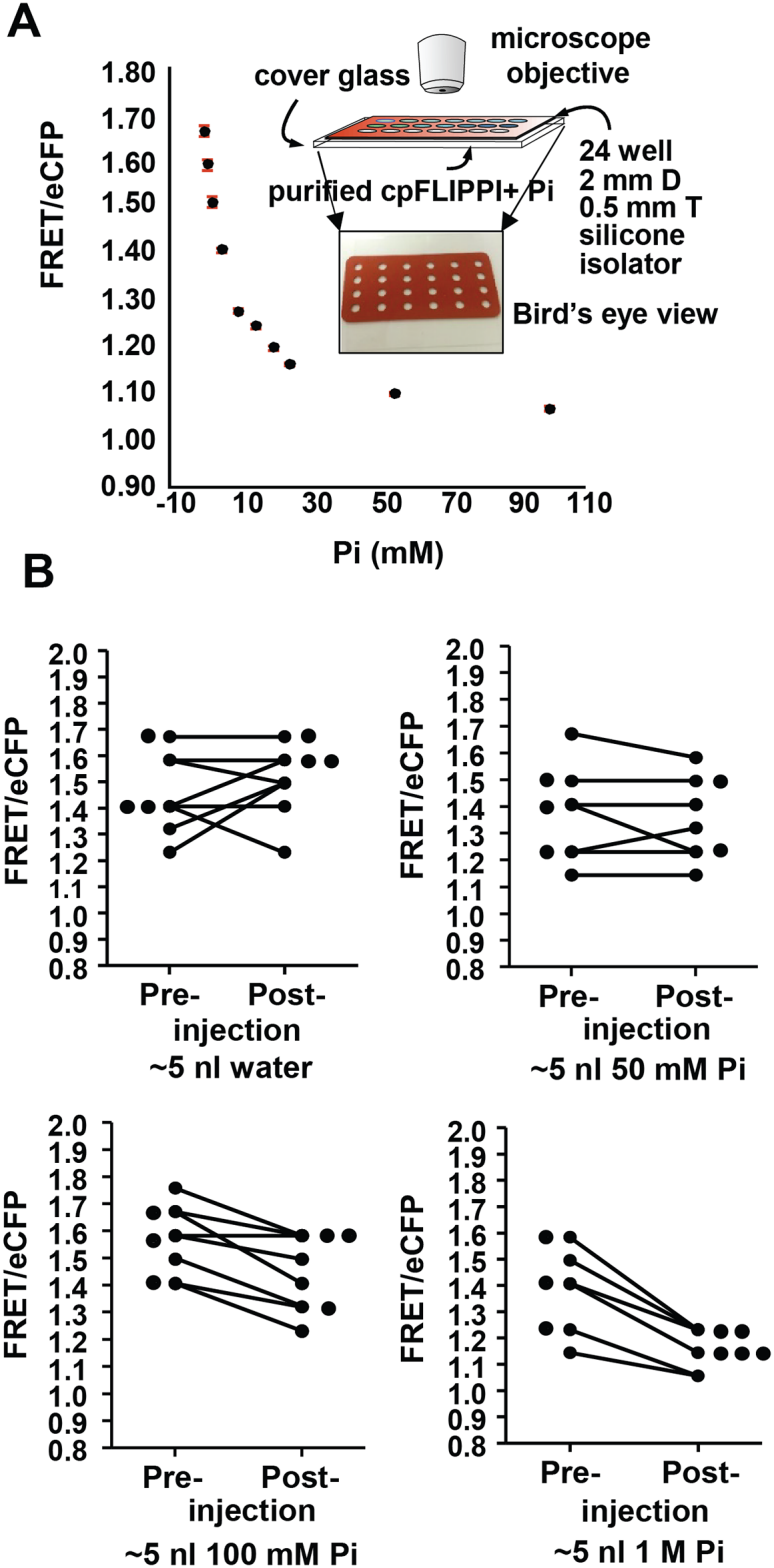
Direct Pi-induced changes in FRET response of cpFLIPPi-6.4m are demonstrated *in vitro* and *in vivo*. (A) *in vitro* Pi binding curves of cpFLIPPi-6.4m generated by mixing purified sensor protein with different Pi concentrations. Mean and standard deviation values were calculated from three individual wells. Inset shows a cartoon and bird’s eye view of the silicone isolator apparatus for imaging solutions of Pi and the sensor. (B) *In vivo* Pi dependent FRET responses were measured by microinjecting Pi buffer at different concentrations into the intestinal cells. Plots show the effect of Pi injection on individual intestinal FRET ratios of the worms, before and 30 sec after Pi injection. FRET Excitation-445 nm: Emission- 542/27 nm, eCFP Excitation- 445 nm: Emission 483/32 nm.

We wanted to test how well the cpFLIPPi-6.4m sensor could report changes in the concentration of Pi in the cytosol of intestinal cells. However, we first had to determine whether our microscope imaging set-up can measure a dose-dependent Pi response of the sensor and its dynamic range. To estimate the Pi-dependent FRET response pattern of cpFLIPPi-6.4m under the same image acquisition settings as used for *in vivo* measurements, we prepared an *in vitro* calibration using purified cpFLIPPi-6.4m in protein dilution buffer containing different concentrations of Pi. Each solution was placed in a well of a silicone isolator adhered to a glass coverslip (Fig 3A inset), and then imaged under the same camera settings and optics used for *in vivo* measurements. We measured a Pi-dependent FRET response indicative of single-site Pi binding (Fig 3A). This result indicates that the optics of the microscope can report subtle changes in Pi concentration, similar to what we previously reported using a plate reader assay [11].

Considering that the buffer composition in the *in vitro* assay most likely does not precisely mimic the intracellular context, we wanted to determine whether the cpFLIPPi-6.4m could report a dose response to Pi *in vivo*. To determine relative changes in FRET/eCFP ratio with changing *in vivo* Pi levels, we injected 0.5 nl of different concentrations of Pi buffer directly into the intestinal cells of worms. For each worm, FRET/eCFP was measured before and approximately 1 min after injection. We reasoned that to restore homeostasis, the live worm intestine should attempt to remove and/or redistribute some of the excess supplied Pi; however, the injected concentrations should still elicit a cpFLIPPi-6.4m-measurable increase or dilution in the amount of cytosolic Pi. Indeed, we observed a Pi dependent change in FRET/eCFP ratios resulting from the injections (Fig 3B). Some of the worms injected with water exhibited a rise in FRET/eCFP ratio, suggesting a dilution of the internal Pi concentration. However, with injection of ~5 nL of 100 mM and 1 M Pi, the intestinal FRET/eCFP ratio decreased, consistent with an increase in Pi concentration. Interestingly, the FRET/eCFP ratio of the worms injected with 50 mM Pi did not change significantly post injection suggesting that the intracellular Pi concentration in the worms after the injection might be similar to *in vivo* pre-injection Pi levels. We estimated the *in vivo* Pi concentrations after injecting 0.5 nl of 50 mM Pi buffer in the intestine of the worm by calculating the volume of the cylinder-shaped intestine and assuming that the amount of injected Pi distributes and equilibrates throughout the whole volume of the intestine. From these results, we suggest that the sensor can directly measure *in vivo* Pi changes, and that the average concentration of Pi in the intestinal cytosol of the injected hermaphrodites is in the range of ~5.8 mM.

**Fig 4:**
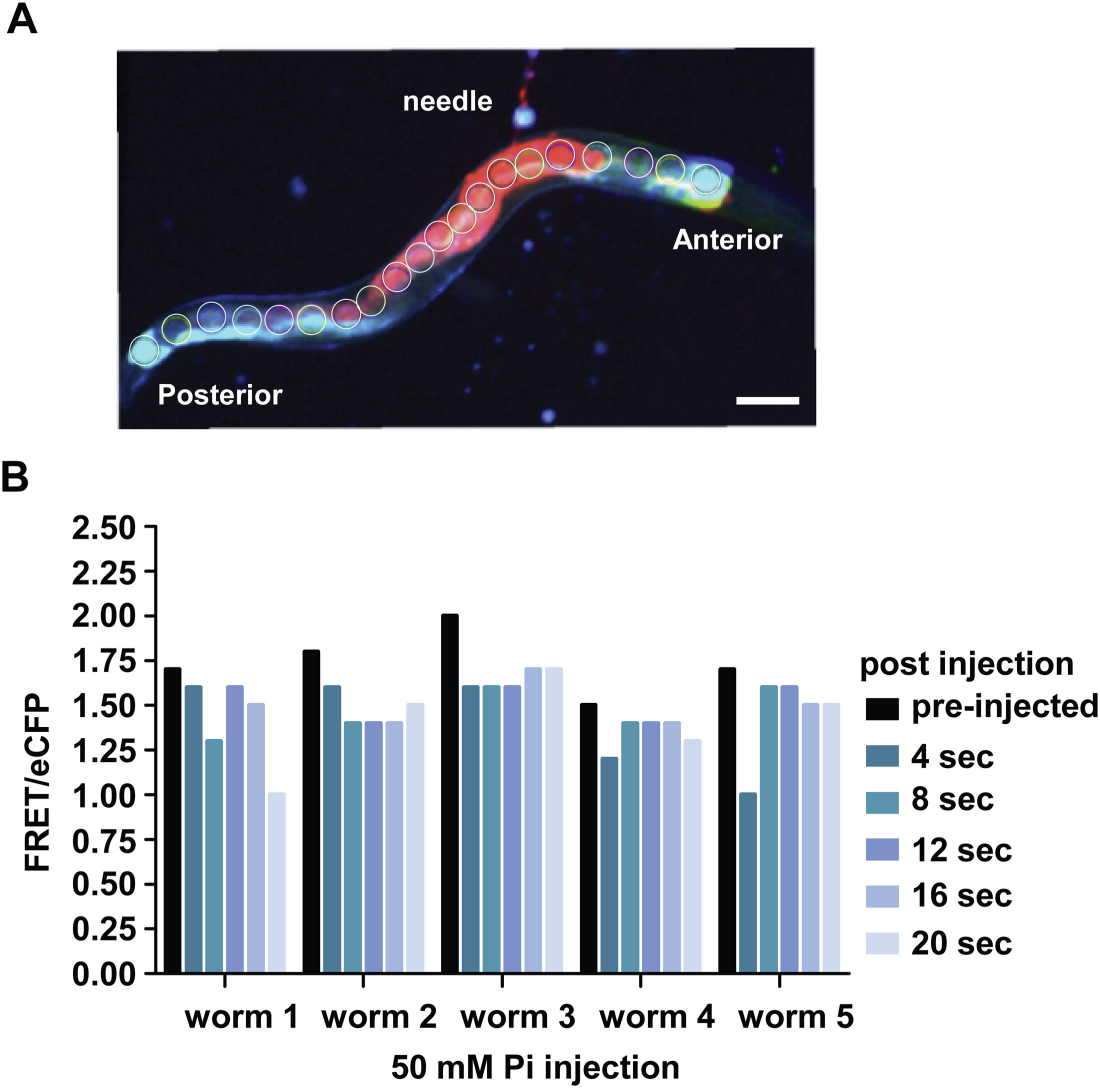
cpFLIPPi-6.4m can report rapid changes in Pi-induced FRET *in vivo*. (A) Image showing spread of the injected fluid along the intestine, visualized by injecting propidium iodide. (B) Rapid decrease in FRET/eCFP ratio after Pi injection into the intestinal cells. Bars represent mean FRET/eCFP ratio taken from ROIs 50–100 μm from the puncture. FRET Excitation-445 nm: Emission- 542/27 nm, eCFP Excitation- 445 nm: Emission 483/32 nm. Scale bar is 100 microns.

We hypothesized that the 50 mM Pi injection did not show a gross change in the average intestinal FRET/eCFP ratio because much of the injected Pi equilibrated within the intestine during the time between injection and image capture. To see whether the sensor could detect rapid small changes in local Pi, we injected a small volume (<0.1–0.2 nl) of 50 mM Pi buffer into an intestinal cell of five different animals. The dye propidium iodide was included in the Pi buffer to aid in monitoring the flow of the buffer within the intestine (Fig 4A). FRET/eCFP was monitored 50–100 microns from the injection site before injection and every 4 sec after injection, for up to 20 sec. Continuous exposure to laser excitation will bleach the fluorophores of the cpFLIPPi-6.4m sensor. Thus we imaged the same cells at 4 second intervals to minimize photo-damage of the cpVenus from the sequential imaging. We observed the FRET/eCFP ratio decreased locally immediately after injection (Fig 4B). Although the FRET/eCFP ratio slightly readjusted afterwards, it remained below the initial value for the duration of the experiment. Taken together, these results indicate that cpFLIPPi-6.4m is capable of reporting small and rapid increases in cytosolic Pi concentration.

### Monitoring the distribution of Pi in live worms

**Fig 5:**
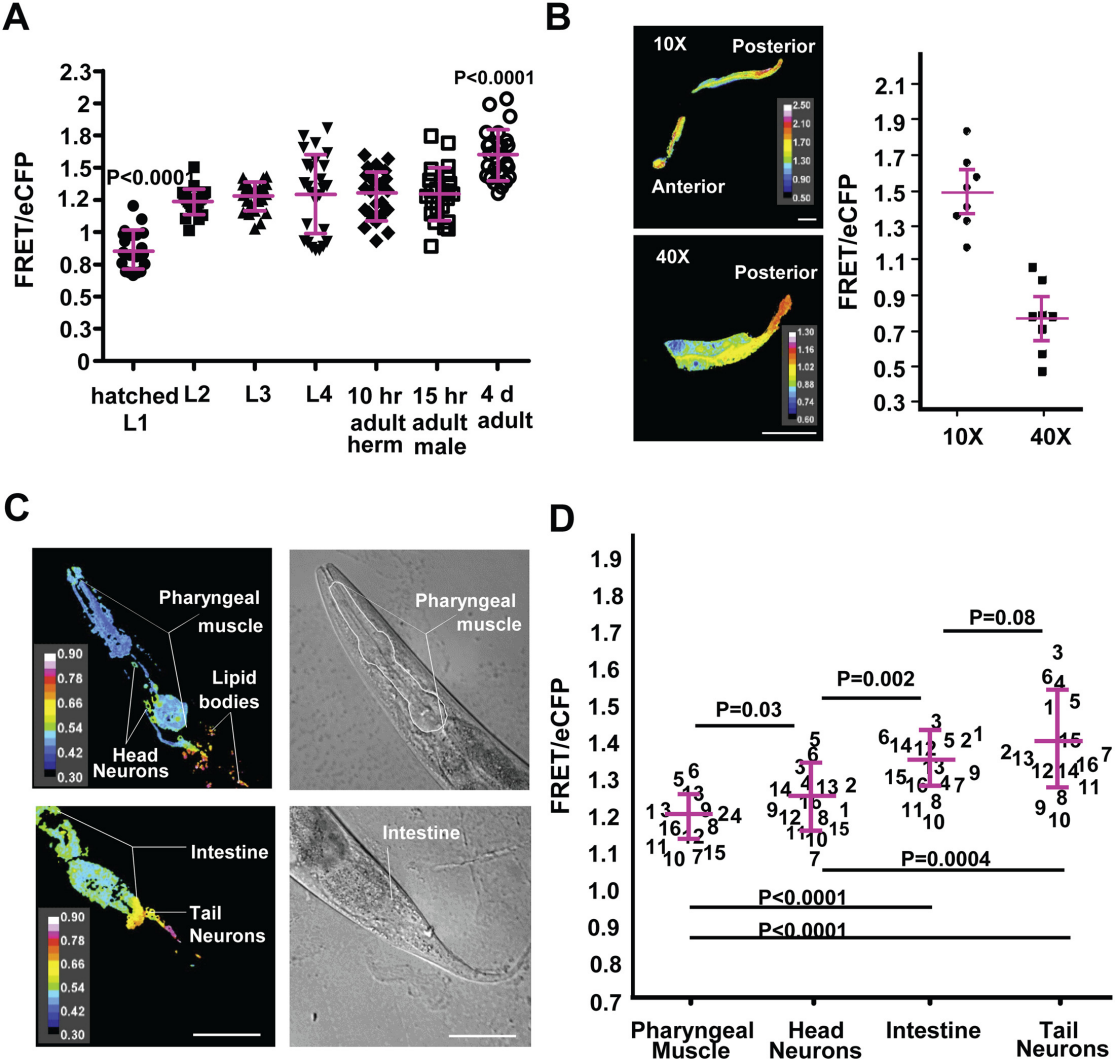
Pi levels differ during development and in different cell types. (A) Pi levels in the intestine of *C*. *elegans* at different developmental stages. Plotted data points represent mean FRET/eCFP ratios of the intestine of individual animals. Shown for each developmental stage is the population FRET/eCFP ratio mean and standard deviation values. (B) Intestinal FRET/eCFP ratio of a representative sample is depicted as a ratio image of FRET/eCFP. Mean FRET/eCFP values of 8 individual animals are plotted in the graph after imaging the same region of the intestine sequentially under 10x and 40x magnification to determine the effect of different image capture settings on the FRET/eCFP ratios. The difference between the mean FRET/eCFP ratios was used as an additive correction factor for all subsequent experiments carried out using the 40x objective. (C) Differential interference contrast micrographs and FRET/eCFP ratio images showing variation in FRET ratios in the pharyngeal muscle, head neurons, tail neurons and intestine of a representative animal. (D) Mean FRET/eCFP ratio values are plotted in the graph as separate distributions for individual cell types of 16 animals. Each animal was given a numerical identifier. FRET Excitation-445 nm: Emission- 542/27 nm, eCFP Excitation- 445 nm: Emission 483/32 nm. Scale bar is 50 micron. P values were obtained from Student’s T test.

To study Pi metabolism *in vivo*, we needed to establish Pi dynamics in the worm with respect to age and developmental stage of the animal. Previous studies have shown changes in concentration of metabolites including organic phospho-compounds during larval development of the animals [33]. However, intestinal Pi concentrations in live animals through different larval stages were unknown. We imaged worms at different developmental stages, including hatched L1, L2, L3, L4, 10 hr adult hermaphrodite, 15 h adult male and 4 d adult hermaphrodites to measure relative FRET/eCFP of the intestine (Fig 5A). The FRET/eCFP ratios we recorded depict a large variation in intestinal Pi in all the stages, essentially creating a range of Pi concentration, which the animals maintain. The mean FRET/eCFP obtained at the different developmental stages suggest that there is an average decrease in intestinal Pi as the worm matures beyond the hatched L1 stage. A possible explanation of this could be that the hatched L1 larvae still had abundant nutrients carried over from their embryonic stage, and may not have assimilated the free Pi into other macromolecules. In contrast, the range of Pi concentrations in the intestine was at relatively similar levels during the developmental stages of L2 through 15 h adult worms. 4 d old animals had lower levels of Pi in the intestine compared to other stages. We observed similar levels of sensor protein expression in the different developmental stages of the worms as measured by arbitrary fluorescence units (A.U) of eCFP in the following populations (mean +/- SD; n = 23–25 for each population); L1: 3715 +/-2677, L2: 4426 +/-841, L3: 3678 +/-844, L4: 3515 +/-1079, Adult: 5705 +/-2945, 4 day old adult: 6972+/-3344 A.U. We have already demonstrated that FRET/eCFP ratios were constant over a 4-fold variation in fluorescent intensity values of the donor (Fig 1D). Thus these data strongly suggest that the variations in FRET/eCFP ratio were due to differences in Pi levels and not a consequence of different levels of sensor protein in the intestine of these animals. These findings suggest that Pi levels in the intestine of the worm can vary at the two extreme ends of the development series; however, during the major part of their life span the animals maintain similar Pi levels in their intestine.

After establishing the range of Pi levels in the intestine of the worms, we studied the patterns of Pi distribution in other cells and tissues of the animal. Since the intestine is the first organ for Pi entry and subsequent dissemination, we wanted to determine if the cytosolic Pi concentrations in different cells of the animal were similar to those in the intestine. To achieve this, we used the heat-inducible *hsp-16* promoter to express the cpFLIPPi-6.4m sensor throughout the body of the animal. This construct allowed us to determine FRET/eCFP ratios in different cell types, including head neurons, tail neuron, pharyngeal muscle and intestine. However, the time we used for the heat shock promoter-expressed cpFLIPPi to accumulate in reasonable amounts for these cells was not sufficient to provide a strong signal in other large cell types, such as the syncytial hypodermal cells. Unlike the intestinal cells, many of the other cells of *C*. *elegans* must be viewed with higher magnification. Unexpectedly, we found that FRET/eCFP ratios differed with 10x and 40x magnifications (Fig 5B). This difference was a constant factor so an additive correction allowed comparisons of FRET/eCFP ratios at the two magnifications. Interestingly, comparisons of FRET/eCFP ratios in different cell types revealed a pattern in which Pi concentrations were highest in the pharyngeal muscles, followed by head neurons, intestine and tail neurons (Fig 5C and 5D). These cell-specific differences in Pi concentrations presumably reflect different physiological functions for each cell type. However, the mechanisms that give rise to these differences remain unknown. Nonetheless, these results helped us establish that the cpFLIPPi-6.4m sensor can distinguish Pi levels in different cell types of an animal and in different developmental stages under defined growth conditions.

### Monitoring metabolic status of cells due to perturbations in the external environment

**Fig 6:**
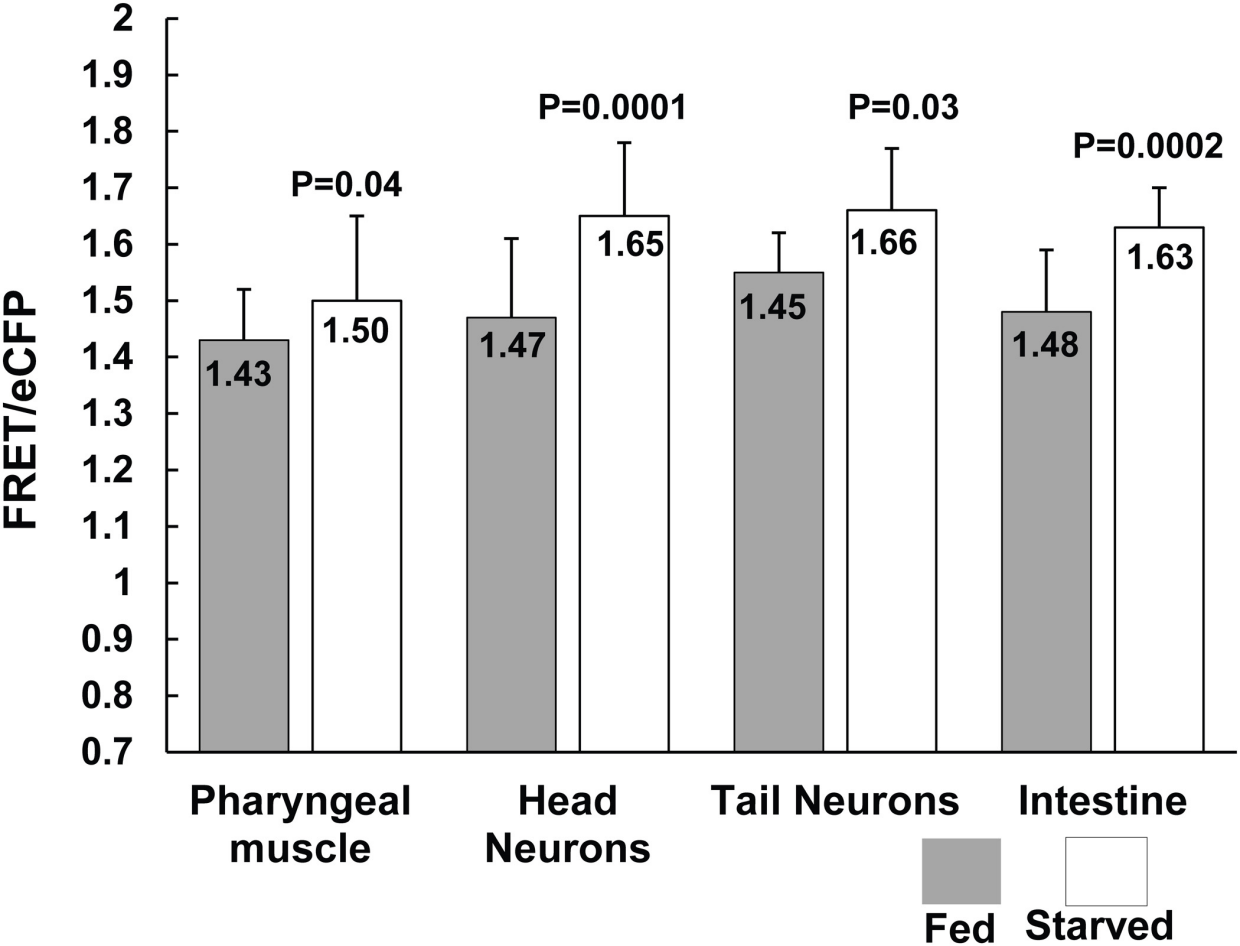
Nutrient starvation alters Pi levels in different cell types of *C*. *elegans*. 24 h adult hermaphrodites were held in NGM plates without bacteria and imaged to compare FRET ratios with the control group, maintained in NGM plates with bacteria. Plotted values are mean FRET/eCFP with standard deviation measured from 13 independent worms. FRET Excitation-445 nm: Emission- 542/27 nm, eCFP Excitation- 445 nm: Emission 483/32 nm. P values were obtained from Student’s T test.

We finally asked if the FRET/eCFP ratio could report the metabolic status of the cells under altered environmental conditions. Under adverse external conditions such as food deprivation, there is no new external source of Pi. Thus, the supply of Pi to different cells of the animal should be limited. To maintain normal metabolic functions, the cells must carry out ATP synthesis; however, when access to Pi is limited by starvation, the cells should rely on recycling internal Pi stores. We predicted the FRET/eCFP ratio should report the altered metabolic status of the cells under starvation as a change in the cytosolic Pi concentration. To measure FRET/eCFP in the pharyngeal muscles, head neurons, tail neurons and intestine of a food-stressed animal, we first starved 24 hour adult hermaphrodites for 6 hours on NGM plates lacking an OP50 bacterial lawn. We maintained a control group on NGM plates with OP50 bacteria for 6 hours and imaged the worms in the same way. After 6 hours of starvation, the FRET/eCFP ratio increased in all the cell types examined, indicating a decrease in Pi levels (Fig 6). However, the effect of starvation on Pi concentration was not same in all of the cell types. The relative decrease in Pi was greatest in the intestine and head neurons of the worms, followed by the tail neurons and pharyngeal muscle. Hence, we inferred that nutrient starvation causes a decrease in cytosolic PI concentrations in different cell types of the worms by different magnitudes, suggesting their varied metabolic functions, Pi demand and utilization.

**Fig 7:**
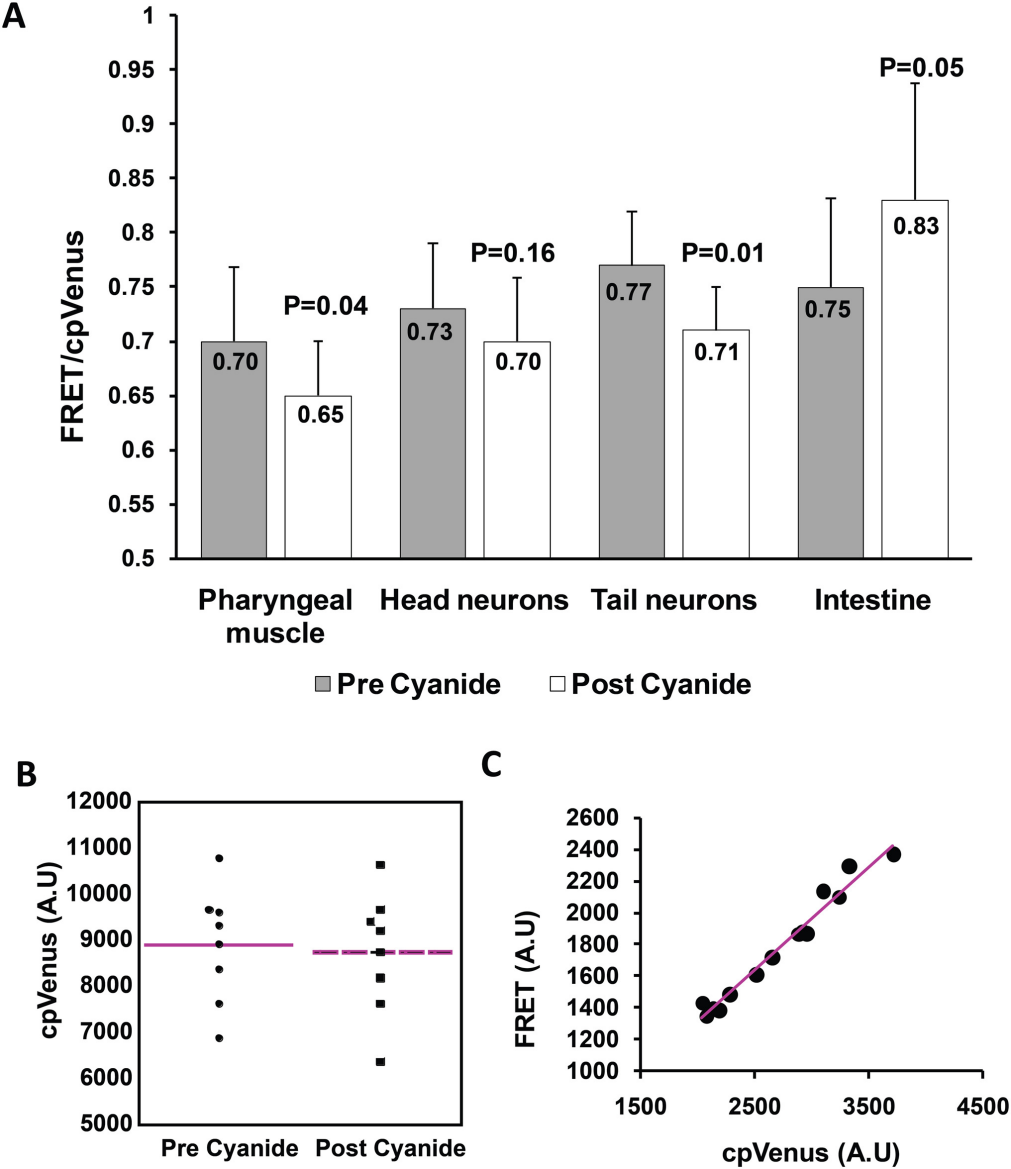
Cyanide treatment alters Pi levels in different cell types of *C*. *elegans*. (A) 24 h adult hermaphrodites were imaged prior to and after treatment with 10 mM sodium cyanide to study the effect of cyanide on cytosolic Pi levels. Plotted values are mean FRET/cpVenus with standard deviation measured from 11 independent worms. P values were obtained from Student’s T test. (B) Graph shows the effect of cyanide on fluorescence intensity of purified sensor protein. cpVenus emission was measured with purified sensor protein in silicone well isolators prior to and after treatment with 10 mM sodium cyanide. Plotted data represent mean cpVenus intensity from 8 independent wells. (C) Plot shows the effect of cyanide on FRET/cpVenus ratios of *C*. *elegans* intestinal cells. The FRET/cpVenus ratio was calculated from multiple ROIs in the intestine of a worm after treating it with 10 mM sodium cyanide. The linear relationship between FRET and cpVenus shows that the ratio FRET/cpVenus is unaffected by cyanide. FRET Excitation-445 nm: Emission- 542/27 nm, cpVenus Excitation- 515 nm: Emission 542/27 nm.

Since a limitation in Pi supply resulted in decreased cytosolic Pi concentrations, we wanted to determine if inhibition of respiration could increase Pi levels. Metabolic inhibitors such as cyanide inhibit ATP synthesis during aerobic respiration [34]. Since the living cells still have to hydrolyze ATP, and thereby liberate Pi to perform cellular functions, we predicted that cyanide treatment should result in an accumulation of free Pi in the cell cytosol. We treated 24 hour old hermaphrodites with 10 mM sodium cyanide and imaged them prior to and 1.5 to 2 min after the cyanide treatment to measure the FRET/eCFP ratio (Fig 7A). We optimized the imaging time based on the time it took for the worms to reduce their locomotion. Unexpectedly, the cyanide treatment affected the *in vivo* fluorescent properties of the sensor, resulting in a general decrease in fluorescent intensity of cpVenus and to a lesser extent eCFP. This was not a direct effect of sodium cyanide on the sensor protein, because it had no effect on FRET/eCFP ratios when added directly to purified sensor protein in *in vitro* Pi binding assays (Fig 7B). Thus we hypothesize that the indirect effect on the fluorophores was due to alterations in the cellular intracellular environment, aggravated by the cyanide treatment. This effect complicated our FRET/eCFP ratio analyses, since the changes in eCFP emission, due to Pi-mediated FRET energy transfer, might no longer be related to the cyanide-induced reduction of the FRET-derived cpVenus emission. To work around this issue, we instead calculated the FRET ratio by dividing FRET by the cpVenus emission. This method is not as sensitive as dividing FRET by eCFP because the magnitude of the FRET/cpVenus ratio is smaller than FRET/eCFP ratio. However, since this ratio incorporates the emission of the same fluorophore, we reasoned that the effect of cyanide should reduce the fluorescence intensity of the 445-excited FRET and the standard 515-excited emission proportionately, thus allowing the 515-excited emission to be a reference for FRET changes. Indeed, we found that over a range of fluorescence intensities, the plot of FRET/cpVenus is linear (Fig 7C) suggesting this normalization method corrects any adverse effect of cyanide in the resultant FRET ratio. Using this method, we found cyanide decreased the FRET/cpVenus ratio in the pharyngeal muscle, head neurons and tail neurons of the worms (Fig 7A). The decrease in FRET/cpVenus ratio is as predicted, since physiological ATP hydrolysis, coupled with cyanide-attenuation of ATP synthesis should increase cytosolic Pi. However, the intestine showed the opposite response, suggesting that cyanide treatment caused a decrease in intestinal Pi content.

**Fig 8:**
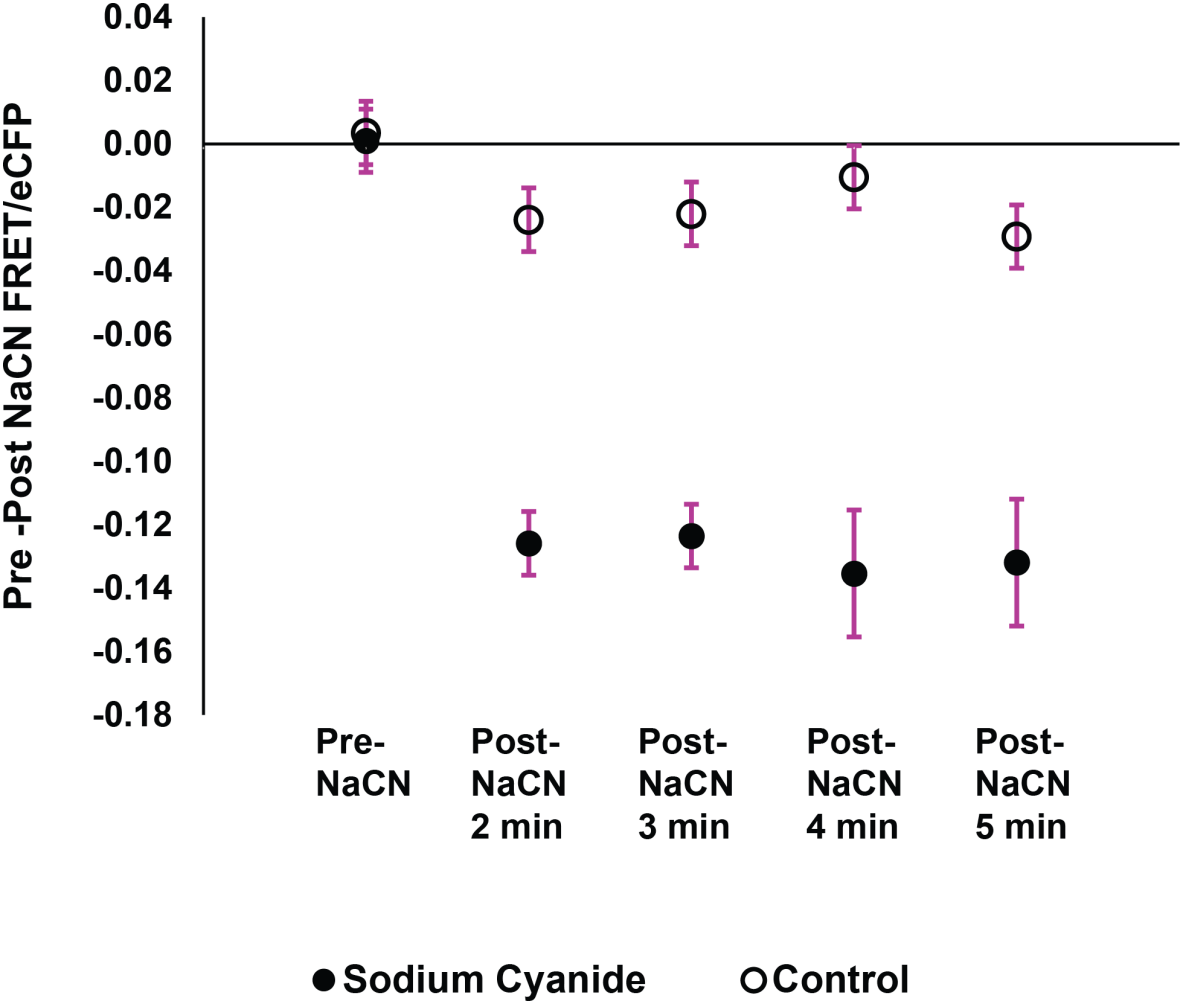
Cyanide causes expulsion of intestinal Pi. Temporal profile of Pi expulsion from the intestine after cyanide treatment. Protein solution in the silicone well was measured at 1 min intervals up to 5 min after treating the suspended worms with 10 mM sodium cyanide. The maximum decrease in FRET/eCFP ratio is observed after 2 min then remains stable. The control group was treated with extra sensor protein solution instead of 10 mM sodium cyanide. Data shows mean and standard error values of measurements taken from three individual wells.

One possible reason for the lower intestinal Pi content is that cyanide induces excretion of intestinal contents, including phosphate. To test this possibility we treated 35–40 worms with 10 mM sodium cyanide solution while holding them in an external buffer solution containing 1.7 μM cpFLIPPi sensor protein. We reasoned that if the worm's intestinal cells excreted their excess cytosolic Pi, then the FRET/eCFP ratio calculated from imaging the external sensor-buffer solution would decrease. We added the worms to a total liquid volume of 5 μl in a silicone isolator well. We then imaged the protein solution in the well prior to and after treating the worms with sodium cyanide. We found that after the cyanide treatment, the FRET/eCFP ratio of the well decreased, indicating increased Pi in the well (Fig 8). The decrease in FRET/eCFP was maximal after 2 min of cyanide treatment and stabilized thereafter, consistent with the idea that the Pi in the well was released from the intestine of the worms, rather than from continual breakdown of the animal’s cellular components. This effect was not observed in negative controls where the worms were treated with extra sensor protein solution instead of sodium cyanide. These results support the hypothesis that cyanide treatment causes increased cytosolic Pi levels due to inhibition of respiration in certain cells of the animal, while the intestine (and possibly other cell types that we did not image) undergoes secretion of contents, which includes Pi.

## Discussion

Measuring the amount of Pi present in a tissue or cell extract is straightforward with current analytical methods, but novel tools and methods are needed to track changes in Pi concentrations within individual cells of a live animal. In this study, we explored the utility of ratiometric imaging of a genetically encoded FRET-based Pi sensor, cpFLIPPi-6.4m, for monitoring cellular Pi concentrations in *C*. *elegans*. Demonstrating the efficacy of the sensor in the context of a live animal was a critical first test. The relatively large and interconnected cells of the intestine were ideal targets. Indeed, changes in FRET/eCFP ratios after injection of Pi solutions or water directly into intestinal cells confirmed that the cpFLIPPi-6.4m sensor is responsive to both increased and decreased Pi concentrations within the worm's intestinal cellular environment.

Endogenous intestinal Pi concentrations varied throughout development with a consistent pattern. Specifically, Pi concentrations were greatest in freshly hatched L1 larvae, which may reflect a large maternal supply of Pi, then decreased in L2 and remained relatively constant through the young adult stages, then decreased further in older adults. This cellular pattern largely mirrors that of overall metabolic status as previously defined from studies of oxygen consumption rates and ATP accumulation [35, 36]. That is, as the developing larva metabolizes its food via oxidative processes, intracellular Pi concentrations are expected to decline due to incorporation into organic molecules, including ATP. This situation must differ in older adult worms because their metabolic rates are lower than during the larval stages [33], yet they have significantly less free Pi. It is unknown, but possible, that older adult animals lose a large fraction of intestinal Pi through excretion. Although our data show that intestinal Pi concentrations decline as the population ages, there is also a large range of concentrations in each developmental stage. This biological variation suggests that intestinal Pi levels are not tightly fixed, but are instead maintained within a range, presumably to account for variation in factors such as developmental progression, behavioral activity, feeding and reproduction.

One can attempt to estimate absolute Pi concentrations corresponding to *in vivo* FRET/eCFP ratio values by directly cross-referencing the *in vitro* calibration curve (Fig 3A). For example, the average intestinal FRET/eCFP ratio value for adult animals was 1.4 (Fig 3B), which corresponds to ~5.0 mM Pi based on the *in vitro* calibration curve. This simple comparison, however, can be problematic because chemical environment can affect fluorescence outputs of the eCFP and cpVenus components of the sensor, and it is difficult to predict how closely the environment of the *in vitro* assay solution mimics that of the cytosol. Nevertheless, the concentration estimated from the *in vitro* calibration is in remarkably close agreement with the independent estimate of ~5.8 mM derived from Pi injection experiments, where the final Pi concentration was calculated from the volume of the intestine and the amount of Pi injected that caused no gross change in the FRET/eCFP ratio (Fig 3B).

**Fig 9:**
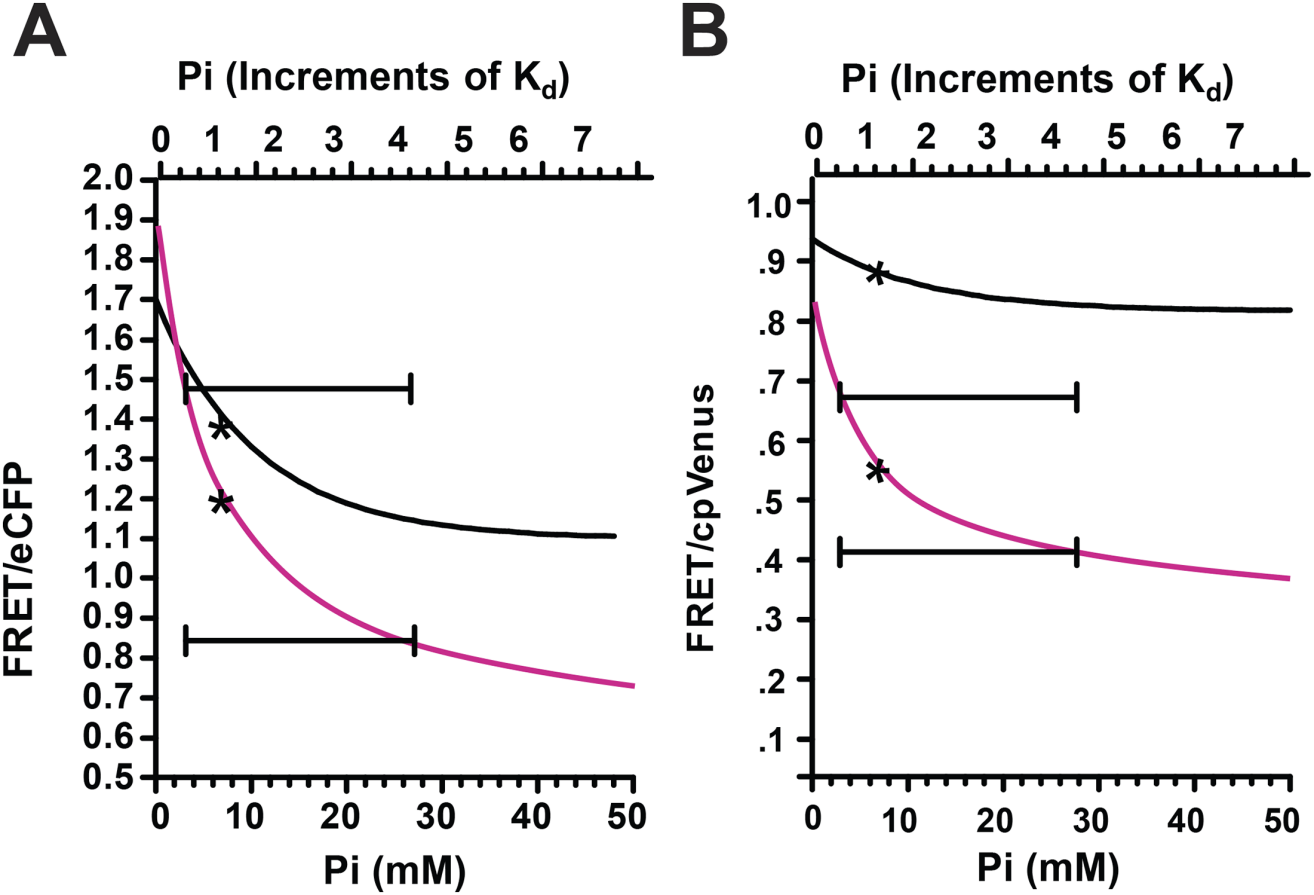
*In vivo* Pi response models generated from the *in vitro* Pi binding curves. The black lines represent the experimentally generated dose dependent Pi response curve of purified cpFLIPPi-6.4m protein plotting Pi concentration against either (A) FRET/eCFP or (B) FRET/cpVenus. The curves follow the binding equation: R-R_o_ = (R_max_-R_o_)*L)/(K_d_+L). R is the FRET ratio; R_o_ is the FRET ratio with no ligand (Pi); R_max_ is the FRET ratio at ligand saturation; L is ligand (Pi); K_d_ is the apparent dissociation constant. From the FRET/eCFP curve, R_o_ is 1.7, R_max_ is 1.1. From the FRET/cpVenus curve, R_o_ is 1.0, R_max_ is .88. The apparent K_d_ for both curves is 6.4 mM. The magenta lines represent the model of *in vivo* Pi dependent response curves generated by substituting the R_o and_ R_max_ for (A) FRET/eCFP or (B) FRET/cpVenus tail values measured from *in vivo* L1 larva and old adult animals. The K_d_ was kept the same as the *in vitro* curve. The asterisk represents the K_d_. The capped lines represent the Pi range between 0.25x and 4x of the K_d_.

The Pi binding dissociation constant imposes an inherent limitation on the range of Pi concentrations that can be probed with the cpFLIPPi-6.4m sensor. That is, it is not possible to accurately resolve concentrations that are far below and far above the K_d_. This limitation exists for all non-linear binding assays. Moreover, our *in vivo* measurements across the different developmental stages (Fig 5A) revealed individuals that display FRET/eCFP ratios outside the extremes of the *in vitro* Pi calibration curve, especially in the hatched L1s (ratio: 0.7) and the 4-day-old adults (ratio: 2). The discrepancy at the tails of the calibration curve suggests that the chemical environment of the *in vitro* assay solution does not fully mimic that of the cytosol at these extremes. We therefore coupled these extreme *in vivo* data values with the *in vitro* calibration curve to generate a model that encompasses the full range of *in vivo* cellular Pi changes. The *in vitro* FRET/eCFP curve can be described by single-site binding equation: R-R_o_ = ((R_max_-R_o_)*L)/(K_d_+L) where R is the FRET ratio, R_o_ is the FRET ratio with no ligand, R_max_ is FRET ratio at saturation, L is ligand (Pi) concentration, and K_d_ is the dissociation constant. For the *in vitro* calibration (Fig 3A), R_o_ is 1.7, R_max_ is 1.1 and K_d_ is 6.4 mM. If the minimum (ratio: 0.7) and maximum (ratio: 2) FRET/eCFP values measured from *in vivo* samples are substituted for R_o_ and R_max_, respectively, then one can modify the *in vitro* curve to roughly accommodate all data points obtain from the *in vivo* data (Fig 9A). If needed, this data treatment can also easily be used to generate a curve using FRET/cpVenus values (Fig 9B) instead the FRET/eCFP values. However, this model assumes that the Pi binding dissociation constant is the same under both *in vitro* and *in vivo* conditions.

When the average FRET/eCFP value for adult worms (1.4) is applied to the modeled *in vitro* calibration curve (Fig 9), it corresponds to ~5.5 mM Pi, which is nearly identical to the estimates without modeling and also from Pi injection. However, the similarities of these values may reflect the fact that the concentrations are within the resolving range of the sensor, i.e., near the K_d_. Estimates of concentrations far from the K_d_ remain suspect. We propose a conservative approach in which estimates are limited to the concentration range spanning from 0.25x to 4x the K_d_ value (Fig 9). These limits also correspond to 20% and 80% of maximum sensor binding. With these limits in mind, we estimate that the intestinal Pi concentration in newly hatched L1 larva is at least 25 mM, and is no more than 1.6 mM in post-reproductive 4-day-old adults. Sensors with significantly different affinities for Pi could be used to define these extreme concentrations with greater precision [11].

We also observed distinct tissue- and cell-specific patterns in cytosolic Pi concentrations. Since these different sites (head neurons, tail neurons and pharyngeal muscle) must initially rely on the intestine for nutrient supply, the different Pi profiles of the cells, which also varied from the anterior to the posterior of the animal, suggest the existence of complex mechanisms that regulate the transport, assimilation and recycling of Pi. These differences may also reflect distinct capacities to respond to changes in dietary supply. Under laboratory conditions, *C*. *elegans* develop on nematode growth media, which contains ~25 mM Pi. This external Pi supports the growth of the bacteria that the worm eats, but is not used by the worm directly [37]. Instead, *C*. *elegans* obtains its Pi from catabolizing the phospho-compounds of its bacterial food [38, 39]. Consequently, Pi deprivation is coupled with overall nutrient deprivation.

Previous studies have shown that nutrient deprivation promotes physiological changes that allow the organism to cope with this adverse condition. For example, nutrient-deprived animals decrease their normal rate of transcription and translation, and initiate degradation of internal energy stores and macromolecules [40–43]. We found that starving animals for just 6 h was sufficient to reduce cytosolic Pi concentrations in all of the cells examined. However, the magnitude of the reduction differed for each cell type suggesting distinct rates of Pi recycling and/or metabolic activities.

In contrast to the reduction in cytosolic Pi levels associated with nutrient deprivation, exposure to cyanide, a mitochondrial electron transport chain inhibitor, increased Pi concentrations in head and tail neurons and in pharyngeal muscles. This increase was expected given the inability to recycle Pi into mitochondrial-produced ATP in the presence of cyanide. Similar effects on cellular Pi have been observed from NMR studies for anoxia, azide and cyanide treatments [44], but the changes in Pi concentrations reported in these studies represent bulk changes due to the lack of cellular resolution. Interestingly, cyanide treatment reduced Pi concentrations in the intestines. Our results suggest that the reduction is due, at least in part, to excretion of Pi to the external environment. These findings indicate that, in spite of internal recycling mechanisms to maintain cellular Pi levels, the intestinal cytosol is sensitive to external perturbations, and that the cpFLIPPi-6.4m sensor can, within limits, resolve subtle changes in Pi as a read-out of the cell’s reaction to these perturbations.
